# Reductive evolution in outer membrane protein biogenesis has not compromised cell surface complexity in *Helicobacter pylori*

**DOI:** 10.1002/mbo3.513

**Published:** 2017-10-21

**Authors:** Chaille T. Webb, Dilini Chandrapala, Siti Nurbaya Oslan, Rebecca S. Bamert, Rhys D. Grinter, Rhys A. Dunstan, Rebecca J. Gorrell, Jiangning Song, Richard A. Strugnell, Trevor Lithgow, Terry Kwok

**Affiliations:** ^1^ Infection & Immunity Program Biomedicine Discovery Institute and Department of Microbiology Monash University Clayton Australia; ^2^ Infection & Immunity Program Biomedicine Discovery Institute and Department of Biochemistry and Molecular Biology Monash University Clayton Australia; ^3^ Department of Biochemistry Faculty of Biotechnology and Biomolecular Sciences Universiti Putra Malaysia Serdang Selangor Malaysia; ^4^ Enzyme and Microbial Technology Research Center Universiti Putra Malaysia Serdang Selangor Malaysia; ^5^ Monash Centre for Data Science Faculty of Information Technology Monash University Melbourne Australia; ^6^ Department of Microbiology & Immunology University of Melbourne Parkville Australia

**Keywords:** BAM complex, beta‐barrel, Helicobacter, outer membrane, surface protein

## Abstract

*Helicobacter pylori* is a gram‐negative bacterial pathogen that chronically inhabits the human stomach. To survive and maintain advantage, it has evolved unique host–pathogen interactions mediated by *Helicobacter*‐specific proteins in the bacterial outer membrane. These outer membrane proteins (OMPs) are anchored to the cell surface via a C‐terminal β‐barrel domain, which requires their assembly by the β‐barrel assembly machinery (BAM). Here we have assessed the complexity of the OMP C‐terminal β‐barrel domains employed by *H. pylori,* and characterized the *H. pylori* BAM complex. Around 50 *Helicobacter*‐specific OMPs were assessed with predictive structural algorithms. The data suggest that *H. pylori* utilizes a unique β‐barrel architecture that might constitute *H. pylori*‐specific Type V secretions system. The structural and functional diversity in these proteins is encompassed by their extramembrane domains. Bioinformatic and biochemical characterization suggests that the low β‐barrel‐complexity requires only minimalist assembly machinery. The *H. pylori* proteins BamA and BamD associate to form a BAM complex, with features of BamA enabling an oligomerization that might represent a mechanism by which a minimalist BAM complex forms a larger, sophisticated machinery capable of servicing the outer membrane proteome of *H. pylori*.

## 1 INTRODUCTION

In the model of Darwinian evolution, bacterial pathogens compete vigorously with normal flora, other pathogens, and host defenses to establish themselves within specific host niches. In most cases, the chain of transmission requires that bacterial pathogens must also adapt to survival in environments aside from the host (Arnold, Jackson, Waterfield, & Mansfield, 2007; Didelot, Walker, Peto, Crook, & Wilson, 2016; Lood, Waldetoft, & Nordenfelt, 2015; Omsland & Heinzen, 2011). These distinct stages in the pathogenic lifecycle generally require remodeling of transcriptional programs, the bacterial surface proteome and metabolic capacity. In many pathogens, such as the various pathovars of *Escherichia coli*, the outer membrane proteome complexity is well characterized and shown to be expanded through variable surface carbohydrates (LPS and capsules) and the acquisition of protein secretion systems, fimbriae, and other cell surface structures through horizontal gene transfer (Diaz‐Mejia, Babu, & Emili, 2009; Hacker & Kaper, 2000; Needham & Trent, 2013). These diverse cell surface structures are topologically intricate, but the majority of protein components are efficiently assembled into the *E. coli* outer membrane by multicomponent molecular machines (Grijpstra, Bos, & Tommassen, 2013; O'Neil, Rollauer, Noinaj, & Buchanan, 2015; Selkrig, Leyton, Webb, & Lithgow, 2014). These complex machines include the β‐barrel assembly machinery (BAM) complex, and the translocation assembly module (TAM), as well as a series of molecular chaperones and other factors in the periplasm (Goemans, Denoncin, & Collet, 2014; Lyu & Zhao, 2015).

Unlike most pathogens, the Epsilon‐proteobacterium *Helicobacter pylori* establishes lifelong infection of the human stomach after acquisition (Dunn, Cohen, & Blaser, 1997), and has coevolved with the human species from before our migration out of Africa (Linz et al., 2007). In its evolution to succeed in this hostile environment, *H. pylori* has undergone a substantial genome reduction: both in gene number (Alm et al., 2000; Baltrus et al., 2009; Tomb et al., 1997) and in the capacity for metabolic reactions it no longer needs (Liechti & Goldberg, 2012; Thiele, Vo, Price, & Palsson, 2005). Recent comparative analyses of strains from distinct human populations revealed that the genome size of *H. pylori* strains from East Asia have decreased compared with the genomes of *H. pylori* from Africa and Europe (Dong et al., 2014; Haley & Gaddy, 2015), highlighting the ongoing evolution toward a minimal genome in this pathogen.

Specific host‐cell interactions and evasion of host defenses are common themes among successful pathogens such as *H. pylori* which makes use of cell‐surface associated and secreted virulence factors to colonize and persist in the gastric environment. The binding specificities of several *H. pylori* cell surface adhesins have been elucidated to date: BabA and SabA bind specific carbohydrate moieties present on gastric epithelial cells (Ilver et al., 1998; Mahdavi et al., 2002; Oleastro & Ménard, 2013); AlpA and AlpB bind laminin found in the extracellular matrix of gastric tissue (Senkovich et al., 2011); and HopQ binds several different members of the family of carcinoembryonic antigen‐related cell adhesion molecules (CEACAM1, 3, 5, and 6) on gastric epithelial cells (Javaheri et al., 2016; Koniger et al., 2016). Exactly how these OMP adhesins are assembled into the outer membrane remains unknown.

Comparative genomics suggest that *H. pylori* possess a limited set of protein secretion systems that enable the presentation of proteins onto the cell surface. Function in Type 1 secretion systems have been proposed for three proteins that are TolC‐like and could potentially serve as outer membrane conduits (Holland, Schmitt, & Young, 2005; Liu, Zheng, & Yang, 2008). Another example is the cytotoxin‐associated gene (*cag*) pathogenicity island‐encoded Type 4 secretion system that translocates the effector protein CagA into gastric epithelial cells (Fischer, 2011), which must also itself be assembled. The VacA toxin that promotes vacuole formation and disruption of mitochondria, has been confirmed as a Type 5 secretion system (Palframan, Kwok, & Gabriel, 2012) and three additional proteins ImaA, FaaA, and VlpC are also considered to be “VacA‐like” (Alm et al., 2000; Celik et al., 2012; Tomb et al., 1997), as they appear to remain tethered onto the surface of the bacterial outer membrane after secretion (Radin et al., 2013).

In keeping with this limited protein secretion capability, *H. pylori* has also dispensed with components involved in OMP assembly, such as the translocation assembly module (TAM) (Heinz, Selkrig, Belousoff, & Lithgow, 2015). The TAM is a membrane embedded protein complex encoded in genomes of all classes of proteobacteria, including most other species of epsilon‐proteobacteria that assists in the assembly of outer membrane proteins that contain complicated topological features, and is essential for virulence‐associated outer membrane protein assembly in other lineages of proteobacteria (Heinz et al., 2015; Selkrig et al., 2012, 2014). In the absence of TAM, how then does *H. pylori* target and assemble its outer membrane β‐barrel proteins? One prospect is that the outer membrane proteome is greatly simplified because of the minimal assembly machinery, although proteomics studies would argue against such simplification, with around 50 outer membrane proteins recognized in past work (Carlsohn, Nystrom, Karlsson, Svennerholm, & Nilsson, 2006; Doig & Trust, 1994; Voss, Gaddy, McDonald, & Cover, 2014). An alternate explanation would be that the BAM complex in *H. pylori* has become more sophisticated; additional BAM subunits in *H. pylori* could maintain its capacity to engage a broad range of substrates, but where these components are not homologous to those found in *E. coli* and therefore not easily recognized by sequence analysis of the *H. pylori* genome.

In order to examine both possibilities, we performed a detailed analysis of *H. pylori* OMP β‐barrel‐complexity together with characterization of the *H. pylori* BAM complex. Renewed sequence characterization of the *H. pylori* type strain 26695 outer membrane proteome revealed reduced diversity of the β‐barrel domain used in outer membrane proteins. This domain is a specific feature recognized and folded by the BAM complex. The reduction in sequence diversity in β‐barrel protein substrates is consistent with a simplification of the outer membrane biogenesis machinery. We found that the BAM complex, far from being more sophisticated, contains just two of the five characteristic components: BamA and BamD. It is, however, larger in size compared to other BAM complexes like *E. coli*. Biochemical characterization of BamA suggests *H. pylori* utilizes a unique oligomerization ability that is not seen in any other characterized BAM complex. Additionally, under the evolutionary pressure for genome reduction and biochemical simplification, a major innovation has arisen in the “Hp_OMP” domain found at the C‐terminus of 44 of the 64 predicted OMPs that are assembled by this BAM complex. We discuss the analogies between the Hp_OMP family and Type 5 secretion systems, significant in that this would be the first bacterial protein secretion system known to be species‐specific.

## 2 EXPERIMENTAL PROCEDURES

### 2.1 Selection of sequences representing the outer membrane proteome

The proteome of *H. pylori* strain 26695 (NC_000915) was retrieved from GenBank. A set of outer membrane protein sequences (Families 1, 2, and 3) was compiled from those identified by comparative genomics (Alm et al., 2000), mass spectrometry (Carlsohn et al., 2006), and comprehensive biochemical methods (Voss et al., 2014). The autotransporter VacA was also added to the collection (Palframan et al., 2012). A Hidden Markov Model (HMM) called AT47‐bb (Celik et al., 2012) containing only the C‐terminal β‐barrel of known autotransporters was used to search for autotransporter‐like (VacA‐like) proteins in *H. pylori* strain 26695 proteome using the method suggested by Likic, McConville, Lithgow, & Bacic (2010). At this stage of analysis three proteins, HorA, HopG, and FrpB‐2, were removed and not assessed further because their genes contain premature stop codons and as such they are annotated across 2 ORFs (*horA* HP0078/HP0079), as a pseudogene (hopG HP0254), or split between a pseudogene and an ORF (*frpB‐2* HP0915/HP0916). A phylogenetic tree was built using 500 C‐terminal residues of each Hp_OMP domains in MEGA6 (Tamura, Stecher, Peterson, Filipski, & Kumar, 2013) with ClustalW alignment and Neighbor Joining method (partial deletion).

To identify those sequences that could be β‐barrel proteins, BOCTOPUS2 (Hayat, Peters, Shu, Tsirigos, & Elofsson, 2016), PRED‐TMBB2 (Tsirigos, Elofsson, & Bagos, 2016), and Phyre2 (Kelley, Mezulis, Yates, Wass, & Sternberg, 2015) were used. Sequences scoring positive for a prediction of at least two potential transmembrane β‐strands were considered potential β‐barrel membrane proteins and were listed in Table 1 if they contained a positive prediction for a Hp_OMP domain (42 sequences). The remaining sequences are listed in Table 2 (19 sequences).

**Table 1:** Hp_OMP domain outer membrane proteins

Protein group	Gene no.	Gene name	BOCTOPUS2 no. β‐strands a	PHYRE2 barrel type	PRED‐TMBB2 no. β‐strands^a^	PRED‐TMBB2 barrel (Y/N)
Family 1 (Hop and Hor proteins)	0009	*hopZ*	8 (4)	Partial	10	Y
	0025	*hopD*	8 (2)	None	8	Y
	0227	*hopM*	8 (4)	Partial	8	Y
	0229	*hopA*	9 (6)	Partial	8	Y
	0252	*hopF*	7 (6)	Partial	10	Y
	0477	*hopJ*	7–8 (6)	Partial	8	Y
	0638	*hopH/oipA*	7–8 (6)	None	10	Y
	0706	*hopE*	8 (8)	Full	10	Y
	0722	*hopO/sabB*	8 (2)	None	10	Y
	0725	*hopP/sabA*	7–8 (2)	Partial	10	Y
	1243	*hopS/babA*	7–8 (2)	Partial	8	Y
	0912	*hopC/alpA*	3 (2)	Partial	8	Y
	0913	*hopB/alpB*	4 (2)	None	8	Y
	0923	*hopK*	7–8 (10)	Partial	8	Y
	1156	*hopI*	6 (2)	None	10	Y
	1157	*hopL*	7–8 (4)	Partial	12	Y
	0896	*hopT/babB*	7–8 (2)	Partial	8	Y
	1177	*hopQ*	8–9 (4)	Partial	10	Y
	1342	*hopN*	8–9 (4)	None	8	Y
	0317	*hopU/babC*	7–8 (2)	Partial	8	Y
	0127	*horB*	7 (8)	Full	8	Y
	0324	*horC*	8 (8)	Full	6	Y
	1066	*horD*	8 (8)	Partial	6	Y
	0472	*horE*	8 (8)	Full	8	Y
	0671	*horF*	8 (8)	Full	8	Y
	0796	*horG*	8 (8)	Partial	12	Y
	1107	*horH*	8 (8)	Full	8	Y
	1113	*horI*	8 (8)	Partial	14	Y
	1469	*horJ*	8 (8)	Full	10	Y
	1501	*horK*	8 (10)	Partial	10	Y
	1395	*horL*	8 (8)	Full	8	Y
Family 2 (Hof family of outer membrane proteins)	0209	*hofA*	17–18 (16)	Full	22	Y
	1083	*hofB*	18 (14)	Full	24	Y
	0486	*hofC*	16–17 (14)	Full	22	Y
	0487	*hofD*	18 (16)	Full	20	Y
	0782	*hofE*	17–18 (14)	Full	20	Y
	0788	*hofF*	17 (14)	Full	20	Y
	0914	*hofG*	18 (14)	Partial	22	Y
	1167	*hofH*	18 (14)	Full	20	Y
Family 3 (Hom family of outer membrane proteins)	0710	*homA*	7 (4)	None	18	N
	0373	*homC*	7 (4)	None	18	N
	1453	*homD*	7 (4)	None	18	Y

a Output from BOCTOPUS2 showing the number of calculation terrain peaks and the predicted number of β‐strands in parenthesis.

**Table 2:** β‐barrel outer membrane proteins other than the Hop, Hof, and Hor proteins

Protein group	Gene no.	Gene name	BOCTOPUS2 no. β‐strands^a^	PHYRE2 barrel type	PRED‐TMBB2 no. β‐strands^a^	PRED‐TMBB2 barrel (Y/N)
Iron‐regulated TonB‐dependent receptors	0686	*fecA‐1*	21 (18)	Full	24	Y
	0807	*fecA‐2*	21–22 (20)	Full	22	Y
	1400	*fecA‐3*	22 (22)	Full	24	Y
	0876	*frpB‐1*	22 (22)	Full	24	Y
	1512	*frpB‐3*	22 (22)	Full	22	Y
TolC‐like efflux pores	0605	*hefA*	4 (4)	Partial	8	Y
	0971	*hefD*	4 (4)	Partial	4	Y
	1327	*hefG*	4 (4)	Partial	4	Y
Autotrans‐porters	0887	*vacA*	12 (12)	Full	12	Y
	0289	*imaA*	12 (12)	Full	54	Y
	0609	*faaA*	7 (8)	Full	6	Y
	0922	*vlpC*	12 (12)	Full	22	Y
Other β‐barrel proteins	0358		2–3 (0)	None	12	N
	0499	*pldA*	12 (12)	Full	14	Y
	0655	*bamA*	16 (16)	Full	16	Y
	0726		8 (8)	None	10	Y
	0839	*fadL*	13 (12)	Full	14	Y
	1216	*lptD*	20 (18)	Full	24	Y
	1467		8 (8)	Full	8	Y

a Output from BOCTOPUS2 showing the number of calculation terrain peaks and the predicted number of β‐strands in parenthesis.

#### 2.1.1 Strains and growth conditions

*H. pylori* strain 7.13 was routinely cultured on GC agar (Oxoid), supplemented with 10% (v/v) horse serum (Invitrogen), 1x (v/v) vitamin mix, vancomycin (10 μg ml^−1^), and nystatin (10 μg ml^−1^) as described previously (Kwok, Backert, Schwarz, Berger, & Meyer, 2002). *H. pylori* culture plates were incubated under microaerobic conditions in an anaerobic jar with CampyGen sachet (Oxoid) at 37°C. Bacteria were harvested from 48‐hr postinoculated plates with a sterile swab. The cells were gently resuspended in brain heart infusion (BHI) media and washed once with the same media prior to subcellular fractionation.

#### 2.1.2 Subcellular fractionation of *H. pylori*

Cells were pelleted and membranes isolated as previously described (Anwari et al., 2010). Total membranes were stored at −80°C. For sucrose gradients total membranes (~400 μg) were subjected to a six‐step sucrose gradient as previously described (Dunstan et al., 2013). Each fraction was TCA precipitated and resuspended in 50 μl SDS‐loading dye. After boiling for 5 min, samples (15 μl) were separated by SDS‐PAGE using 12% polyacrylamide gels for Coomassie staining and Western blotting.

#### 2.1.3 Blue‐native PAGE

Total membranes (100 μg) from *H.pylori 7.13* and *E.coli BW25113* were resuspended in ACA750 buffer (750 mmol/L n‐aminocaproic acid, 0.5 mmol/L EDTA, 50 mmol/L Bis‐tris pH 7.0) and solubilized in the presence of increasing concentrations of n‐Dodecyl β‐D‐maltoside (DDM), Triton X‐100 or digitonin, spinning gently on a tube rotator (Thermoline Scientific) at 4°C for 40 min. Unsolubilized membranes were removed by centrifugation (20,000*g*, 4°C, 10 min). Recombinant *Hp*BamAPOTRA sample (40 μg) was also assessed by BN‐PAGE. Samples were analyzed by BN‐PAGE on 5–16% (w/v) polyacrylamide gels as previously described (Schagger & von Jagow, 1991).

#### 2.1.4 Coimmunoprecipitation

*H. pylori* total membranes (~800 μg) were solubilized in 1% DDM and incubated with 5 μl rabbit anti‐HpBamA_POTRA_, then combined with 50 μl Protein A/G Plus Agarose beads (Santa Cruz Biotechnology) according to procedures previously described (Anwari et al., 2012).

#### 2.1.5 Mass spectrometry and N‐terminal sequencing

Bands of interest were excised from Coomassie‐stained SDS‐PAGE gels or from a PVDF membrane and analyzed by the Monash Biomedical Proteomics Facility for protein identification. For mass spectrometry experiments, the protein was reduced in 2.5 mmol/L DTT at 50°C for 30 min followed by alkylation with 10 mmol/L iodoacetamide for 30 min in the dark at room temperature. Following alkylation a solution containing 1 μg Trypsin Gold (Promega) in 20 mmol/L ammonium bicarbonate was added and the samples incubated at 37°C overnight. Tryptic digests were analyzed by LC‐MS/MS using the Q‐ORBITRAP (QExactive Plus) mass spectrometer (Thermo Scientific) coupled online with a RSLC nano HPLC (Ultimate 3000, Thermo Scientific). Samples were concentrated on a 100 μm, 2 cm nanoviper pepmap100 trap column with 95% buffer A (0.1% formic acid) at a flow rate of 15 μl min^−1^. The peptides then eluted and separated with a 50 cm Thermo RSLC pepmap100, 75 μm id, 100 Ǻ pore size, reversed phase nanocolumn with a 30 min gradient of 90% buffer A (0.1% formic acid) to 25‐min to 30% B (80% acetonitrile 0.1% formic acid) and to 40%B to 30 min, at a flow rate of 300 nl min^−1^. The eluent is nebulized and ionized using the Thermonano electrospray source with a distal coated fused silica emitter (New Objective) with a capillary voltage of 1900 V. Peptides are selected for MSMS analysis in Full MS/dd‐MS^2^ (TopN) mode with the following parameter settings: TopN 10, resolution 17500, MSMS AGC target 1e5, 60 ms Max IT, NCE 27, and 3 *m/z* isolation window. Underfill ratio was at 10% and dynamic exclusion was set to 15 s. Data from LCMSMS run were exported to Mascot generic file format (*.mgf) using proteowizard 3.0.3631 (open source software, http://proteowizard.sourceforge.net) and searched against an inhouse curated database containing *H. pylori* genomes obtained from Uniprot using the MASCOT search engine (version 2.4, Matrix Science Inc.) with all taxonomy selected. The following search parameters were used: missed cleavages, 5; peptide mass tolerance, ±10 ppm Da; peptide fragment tolerance, ±0.02 Da; peptide charge, 2+, 3+, and 4+; fixed modifications, carbamidomethyl; Variable modification, oxidation (Met).

#### 2.1.6 Recombinant protein expression and purification

*Hp*BamA fragments for recombinant protein expression (Table 3) were amplified from *H. pylori* 7.13 and cloned into pPROExHtb to include a TEV‐cleavable N‐terminal hexahistidine tag. Sequences of primers are outlined in Tables 4. Plasmids were then used to transform *E. coli* C41(DE3) cells. Positive clones were selected and grown at 37°C to an OD = 0.8, after which the temperature was decreased to 18°C and protein expression induced with 0.1 mmol/L IPTG. Cells were grown overnight at 18°C and harvested the next day. The cell pellet was lysed in 20 mmol/L Tris‐HCl (pH 7.5), 400 mmol/L NaCl, 5% glycerol and broken with an Emulsiflex (Avestin). Cell debris was collected by ceintrifugation at 28,000*g* for 15 min at 4°C, and the supernatant applied to a 5 ml HisTrapHP column (GE Healthcare). The protein was eluted with a 20 mmol/L–1 mol/L imidazole gradient, and appropriate fractions pooled and concentrated. The His‐tag was cleaved with TEV prior to loading on a Superdex200 16/600 gel filtration column (GE Healthcare) in 20 mmol/L Tris‐HCl (pH 7.5), 250 mmol/L NaCl, 5% glycerol. Protein was concentrated to ~7 mg ml^−1^, glycerol added to 10% final concentration and aliquots snap‐frozen in liquid nitrogen. Expression and purification of *Ec*BamA was performed in a similar fashion as described above.

**Table 3:** **Protein expression plasmids**

Plasmid	Vector	Insert
*Hp*BamA_POTRA_	pPROExHtb cloned in with EcoRI/XhoI	PCR fragment generated with F_EcoRI_HpBamA_23 and R_HpBamA_469_XhoI
*Hp*BamA_Δ46POTRA_	pPROExHtb cloned in with EcoRI/XhoI	PCR fragment generated with F_EcoRI_HpBamA_23 and R_HpBamA_469_XhoI
*Hp*BamA_Δ67 POTRA_	pPROExHtb cloned in with EcoRI/XhoI	PCR fragment generated with F_EcoRI_HpBamA_67 and R_HpBamA_469_XhoI
*Ec*BamA_POTRA_	pPROExHtb cloned in with NcoI/XbaI	PCR fragment generated with F_NcoI_EcBamA_20 and R_EcBamA_422_XbaI

**Table 4:** Oligonucleotide primer sequences

Primers	Sequence
F_EcoRI_HpBamA_23	5′ gccgaattcggaaaatgacggc 3′
F_EcoRI_HpBamA_46	5′ ccggaattccgaaagaaatgaaagtc 3′
F_EcoRI_HpBamA_67	5′ccggaattccgaaaaatgaagttc 3′
R_HpBamA_469_XhoI	5′ cgactcgagctattacccagtacgccc 3′
F_NcoI_EcBamA_20	5′‐ccgcccatgggagctgaagggttcgtagtg‐3′
R_EcBamA_422_XbaI	5′‐ccgctctagatcagttgcgctcttttaccttg‐3′

#### 2.1.7 Size exclusion chromatography‐coupled multiangle light scattering (SEC‐MALS)

Measurements were carried out by injecting purified proteins (200 μg) onto a Superdex200 10/300 gel filtration column (GE Healthcare) preequilibrated with gel filtration buffer (20 mmol/L Tris‐HCl (pH 8.0), 150 mmol/L NaCl, 5% glycerol). Molecular weight standards for the S200 10/300 column are as follows: 440 kDa, 10.3 ml; 232 kDa, 12.2 ml; 158 kDa, 12.6 ml; 75 kDa, 13.9 ml; 43 kDa, 15 ml. The SEC system was coupled to an 18‐angle, static light scattering detector and a refractive index detector (DAWN HELEOS‐II and Optilab T‐rEX, respectively; Wyatt Technology). Data were collected at 25°C at a flow rate of 0.4 ml min^−1^ and analyzed with the software ASTRA6.1, yielding the molecular mass and mass distribution of the samples.

#### 2.1.8 BS3 cross‐linking experiments

Protein was thawed and dialyzed into 20 mmol/L phosphate (pH 7.5), 200 mmol/L NaCl, 5% glycerol. Cross‐linking experiments were performed with 10 μM *Hp*BamA_POTRA_ protein and 100 μM BS3 (Sigma) in phosphate buffer. The reaction was quenched at specific time points by the addition of 20 mmol/L Tris‐Hcl (pH 7.5), and boiled in reducing SDS‐PAGE loading buffer for 5 min before running samples on 10% SDS‐PAGE.

#### 2.1.9 Antibodies

The DNA fragment encoding the soluble POTRA domain (residues 23–469) of *Hp*BamA (YP_002301301.1) was amplified using *H. pylori* 7.13 genomic DNA as a template and cloned into pET22b (Invitrogen) to include a noncleavable C‐terminal hexahistidine tag. Soluble protein was expressed in BL21(DE3), then purified by affinity and size exclusion chromatography. Recombinant protein was used to generate polyclonal antibodies in rabbits by Davids Biotechnologie (Germany). VacA antisera was made in‐house (Monash University Ethics Approval MARP/2016/003) and DnaK monoclonal antibody was purchased from StressGen.

## 3 RESULTS

### 3.1 Radical simplification of outer membrane β‐barrels

The Hop/Hor, Hof, and Hom families of outer membrane proteins have previously been reported to constitute a major fraction of the *H. pylori* outer membrane proteome (Alm et al., 2000). These proteins are defined by virtue of the conserved “Hp_OMP” domain (Pfam01856) that they contain at their C‐terminus. To explore the complexity of the *H. pylori* OMPs further, and to search for any unidentified candidates, we searched the *H. pylori* 26695 genome for proteins that contain the Hp‐OMP Pfam domain and any other genes annotated as “membrane protein” This list was then analyzed against three parameters: presence of a signal peptide (SignalP4.1; (Petersen, Brunak, von Heijne, & Nielsen, 2011)), presence of a membrane integrated β‐barrel (BOCTOPUS2; (Hayat et al., 2016) and PRED‐TMBB2; (Tsirigos et al., 2016)), and structure prediction (PHYRE2; (Kelley et al., 2015)). The process identified 61 proteins overall that are divided into those that do not contain the Hp‐OMP Pfam domain of which constitute a relatively small set of 19 β‐barrel membrane proteins (Table 2) and those that do contain the Hp‐OMP Pfam domain which include the Hop/Hor, Hof, and Hom OMP protein families (Table 1). Most have been previously identified by Alm et al. but several constitute new additional β‐barrel proteins including identification of PldA and LptD, involved in lipid aspects of outer membrane biogenesis (Table 2).

**Figure 1:**
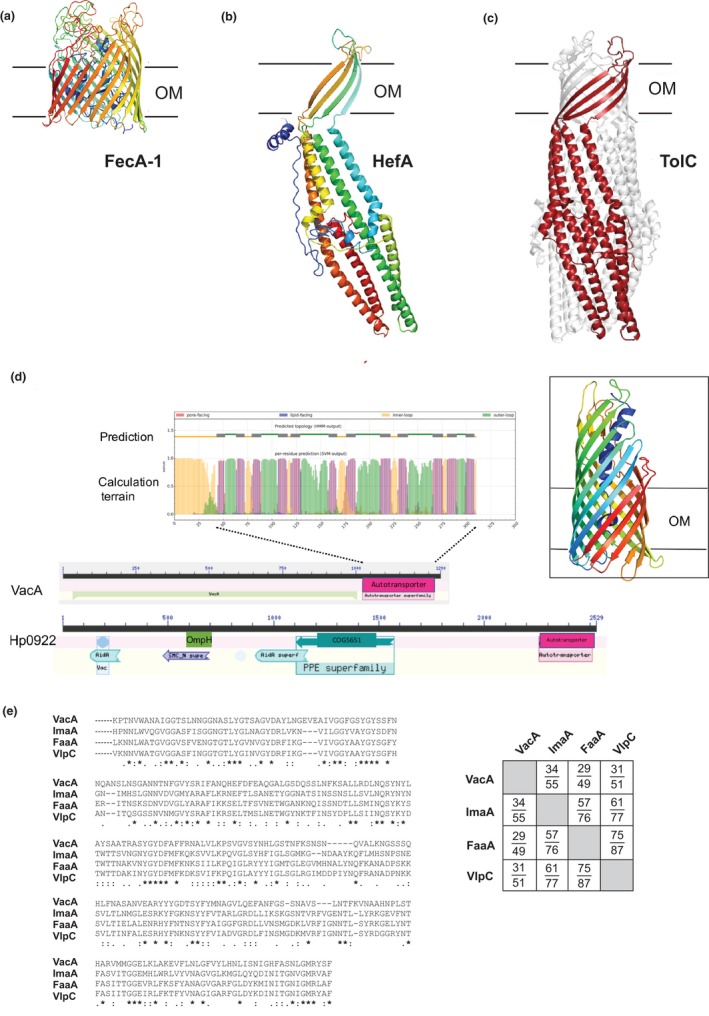
Key elements in the outer membrane proteome of *H. pylori*. (a) Phyre2 prediction of the structure of the FecA‐1 iron transporter. The approximate position of the outer membrane (OM) is indicated. (b) Phyre2 prediction of the transport pore HefA. Only one molecule (i.e., one‐third of the β‐barrel structure) is illustrated. (c) Structure of TolC indicating a monomer (red) in the context of the overall trimeric structure. (d) Conserved domain assessment of the autotransporters VacA and Hp0922/VlpC. The domain signatures in the N‐terminal passenger domain are indicated, and the common β‐barrel domain is indicated in pink. A prediction for a 12‐stranded β‐barrel by BOCTOPUS2 is indicated, where purple shading in the terrain plot represents potential transmembrane β‐strands, with each of these predictions being above the cut‐off score. Magnification of this output is provided in Figure S4. Inset: structure of the 12‐stranded β‐barrel domain of VacA modeled by Phyre2. (e) Multiple sequence alignment of the C‐terminal sequences of VacA, ImaA, FaaA, and VlpC. Asterisks indicate residues conserved in all four sequences; residues conserved in only three (:) or two (.) sequences are also indicated. Inset: the pairwise identities and similarities for these β‐barrel sequences were calculated with EMBOSS Needle. The upper number is sequence identity, whereas the lower number is sequence similarity

Using the structural prediction program Phyre2, many of these *H. pylori* proteins appear consistent with them having structural similarity with characterized homologs in other species. For example, the FecA‐1 protein has the conserved structural features found in other TonB‐dependent ferric transporters (Noinaj, Guillier, Barnard, & Buchanan, 2010) including the plug domain nestled within the barrel domain formed of 22 β‐strands (Figure 1a). The same architecture is predicted for the closely related proteins FecA‐2, FecA‐3, FrpB‐1, FrpB‐2, and FrpB‐3. A second architecture observed was found in the TolC‐like efflux pump, HefA, largely predicted to contain four transmembrane β‐strands and extensive periplasmic α‐helices (Figure 1b), as is the case for the closely related proteins HefD and HefG. By analogy with the structure of *E. coli* TolC (Hinchliffe, Symmons, Hughes, & Koronakis, 2013), it is predicted that three copies of HefA would be assembled into a trimeric protein with a transmembrane domain formed from the resulting twelve β‐strands (Figure 1c).

A twelve‐stranded β‐barrel architecture is predicted for all four autotransporters encoded in the *H. pylori* 26695 genome, the most widely studied being VacA (Figure 1d). Although the other autotransporters are often grouped together with VacA, and annotated as “VacA‐like protein” or “VacA2 protein,” they have distinct passenger domains that are likely to be functionally different proteins within the autotransporter family. Hp0922/VlpC for example has a passenger domain that carries several sequence signatures including COG4372 (DUF3084 domain proteins), OmpH and COG5651 (PPE‐repeat proteins) (Figure 1d). The annotation of “VacA‐like” has arisen because the β‐barrel domains of the four autotransporters are so highly conserved: VlpC has a C‐terminal β‐barrel domain that shares 31% sequence identity with the β‐barrel domain of VacA (Figure 1e), and pairwise sequence identity between the other β‐barrel domains rises to 75% (Figure 1e). In terms of function, they are probably not “VacA‐like”, but they each share what is effectively a single common β‐barrel domain.

The final subset of β‐barrel proteins (Table 2: “other”) contains the outer membrane protein assembly component BamA along with other known membrane proteins such as the lipid transporters LptD and FadL, and the outer membrane anchored enzyme phospholipase A (PldA). Crystal structures of known protein homologues suggest that the β‐strand predictor algorithms applied by BOCTOPUS2 and PRED‐TMBB2 appear in the most part accurate with PRED‐TMBB2 performing the best. A few uncharacterized proteins make up the remainder of this group and these all contain a small number of strands. Despite the range of β‐barrel proteins present in the “other” subgroup, generally the remaining protein subgroups shown in Table 2 exhibit a few, distinct β‐barrel architectures.

### 3.2 The hop, hom, hof, hom proteins

**Figure 2:**
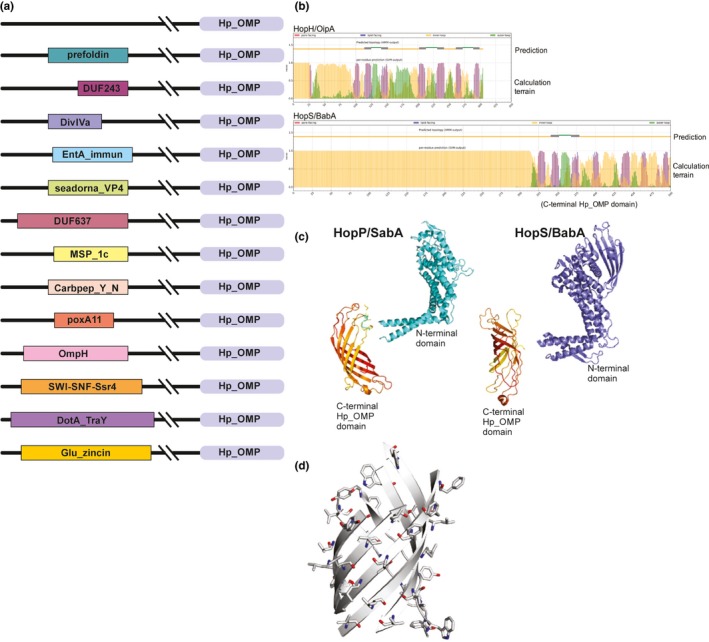
Hop, Hom, Hor proteins, and the Hp_OMP domain. (a) Examples of proteins containing the Hp_OMP domain from H. pylori 26695 genome. The N‐terminal domains vary greatly in size, and sections not carrying recognizable sequence signatures have been deleted. The domain boundaries are otherwise drawn to scale. (b) BOCTOPUS2 prediction of transmembrane β‐strands in HopH/OipA and HopS/BabA. Purple shading in the terrain plot represents potential transmembrane β‐strands, each protein has potentially 7–8 peaks, only some of which score sufficiently high to be counted in the upper topology assessment. BOCTOPUS2 assigns six transmembrane β‐strands to HopH/OipA and two transmembrane β‐strands to HopT/BabB. Magnification of this output is provided in Figure S4. (c) Structure prediction of HopP/SabA and HopS/BabA. The crystal structure of the soluble N‐terminal domain of SabA is depicted in aqua (pdb:4o5j) and the C‐terminal domain is modeled using Phyre2. BabA N‐terminal domain has also been determined by X‐ray crystallography (pdb;4zh0) and the C‐terminal domain predicted using Phyre2. (d) The Phyre2 structural model of HopP/SabA, predicts the C‐terminal domain forms a curved, amphipathic β‐sheet. Side‐chains emanating from the convex surface are indicated, all are hydrophobic residues consistent with residing in a membrane environment. The sidechains emanating from the concave surface (not shown) are predominantly hydrophilic

Updated analysis of the “Hop/Hof,” “Hom,” and “Hor” protein groups with respect to their β‐barrel domains confirmed previous reports that they represent the vast majority of the outer membrane proteome in *H. pylori* 26695, constituting 42 of the predicted 61 outer membrane proteins (Table 1) (Alm et al., 2000). These proteins have been classified into three families based on their C‐terminal β‐barrel domain (Table 1). Family 1 comprising the Hop and Hor proteins, Family 2 containing Hof proteins, and Family 3 contains three related Hom proteins. Despite their separation into families, a common Hp_OMP domain (Pfam01856) at the C‐terminus exists in all three families (Figure 2a). Analysis of the Pfam collection of protein sequences shows that the Hp_OMP domain is confined to *Helicobacter spp*, suggesting a unique, remarkable similarity within these proteins.

While sharing a common C‐terminal domain, these 42 proteins have N‐terminal domains predicted to be structurally and functionally diverse, including experimentally defined roles as adhesins for attachment to host cell surfaces (Zanotti & Cendron, 2014) or porins for metabolite transport (Bina, Bains, & Hancock, 2000). In other members of this family, we find sequence‐based evidence for roles as molecular chaperones, bacteriocins, peptidases, and plasminogen‐binding proteins. In the case of many of these proteins, the N‐terminal domains are substantive in size and have been demonstrated to be extracellular (Voss et al., 2014). Diversity in their sequence and/or function thus exists predominantly within their N‐terminal domain structures predicted to be associated with the C‐terminal Hp_OMP domain (Figure 2a).

This is a feature of broader β‐barrel protein groups such as the Type V secretion system which contains autotransporters (Celik et al., 2012), trimeric autotransporters (Qin, Wang, & Lei, 2015), and inverse‐autotransporters (Heinz et al., 2016).

Family 1 includes the Hop and Hor proteins, where predictions suggest 8–10 β‐strands (TMBB2) or 6 β‐strands (BOCTOPUS2). We note, however, that in several cases (e.g., HopS/BabA) only two or three of these potential transmembrane β‐strands scored better than the cut‐off values used for other bacterial groups (Figure 2b). Phyre2 predictions also ranged in the number of β‐strands that were seen: in cases such as HopP/SabA and HopS/BabA six β‐strands were predicted to form a curved, β‐sheet “part‐barrel” structures (Figure 2c).

Putting to the side the variability in the predicted number of β‐strands, the number predicted is in most cases insufficient for forming a complete β‐barrel domain. One explanation is that the predictor is insufficiently well‐trained for protein sequences from Epsilon‐proteobacteria, and has missed several additional β‐strands. An alternative explanation would be that, like the HefA and TolC proteins, Family 1 proteins oligomerize in order to form a complete β‐barrel. Hydropathy analysis of the predicted structure of Hop/SabA revealed that the side‐chains emanating from the convex surface are predominantly hydrophobic residues, whereas the sidechains emanating from the concave surface are predominantly hydrophilic (Figure 2d). This finding is consistent with them being transmembrane structures, where the convex surface would be embedded in the lipid phase of the outer membrane. Until there are crystal structures available to warrant a stronger conclusion, the topological details of the Hp_OMP domain remains open to interpretation. While a recent study of BabA revealed that it could oligomerize in vivo, apparently forming a trimer and with only the oligomeric form being capable of inducing BabA‐mediated adherence to the gastrointestinal tract (Moonens et al., 2016), trimers of the N‐terminal domain may or may not signify trimerization in the Hp_OMP domain.

The predictions for β‐strands among the Family 2 Hof proteins are stronger, with these proteins predicted to contain many more β‐strands (~14 β‐strands predicted by BOCTOPUS and up to 20 β‐strands predicted by PRED‐TMBB2) which could in principle enable them to form a complete β‐barrel.

The results for Family 3 Hom proteins are more enigmatic. These proteins displayed the largest discrepancy between BOCTOPUS2 and TMBB2 transmembrane β‐strand predictors and in each case a β‐barrel topology was not predicted to be feasible in the structural modeling. This may be indicative of disparities between the *H. pylori* OMPs and the proteins on which the prediction systems were trained, and leaves open the question of whether these proteins can form a “partial” or “full” barrel domain.

**Figure 3:**
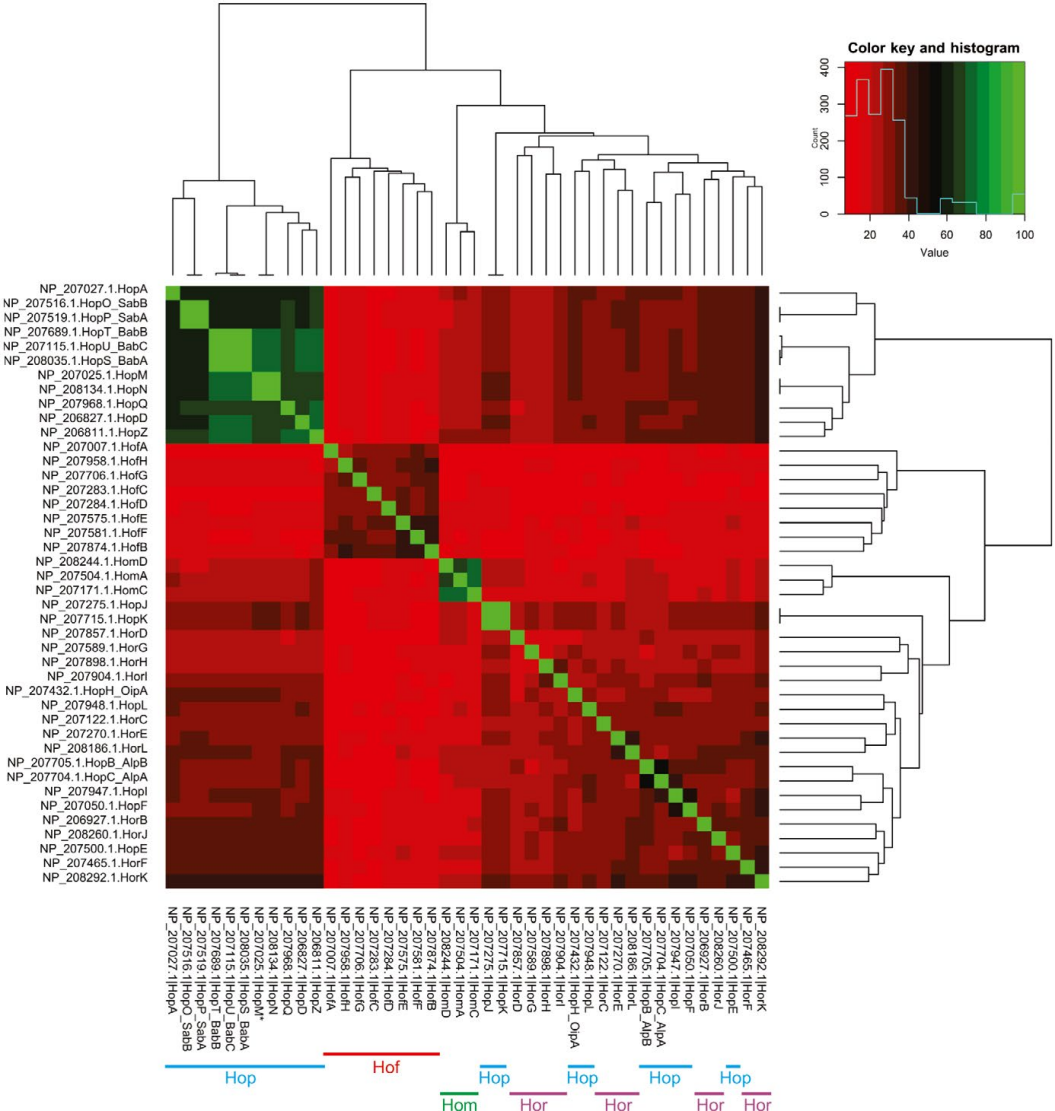
Phylogenetic tree of the barrel domain. Sequence analysis of the 42 proteins carrying the Hp_OMP domain. The heatmap shows the sequences grouped according to sequence similarity, and the red‐green color scale designates the degree of similarity between all 42 sequences. The positioning of the Hop, Hor, Hom, and Hof subfamilies across the matrix is indicated with colored bars

To independently assess the relationships between the Hp_OMP β‐barrel domains, sequence similarity analysis was carried out on the C‐terminal 250 residues of Families 1, 2 and 3. This region of the 42 OMPs is sufficient to encapsulate each protein's entire Hp_OMP domain. Initial characterization by construction of a phylogram confirmed the relationship across these groups, but suggests that the Hop‐Hor classification is an artificial distinction, given that the Hop and Hor proteins are interspersed across the segments of the tree (Figure S1). Inclusion of the C‐terminal 250 residues of the autotransporters suggested only a weak sequence‐based relationship with the Hof proteins in the C‐terminal region. For a more detailed analysis of the Hp_OMP domain relationships, a heat map was calculated (Figure 3). The relationship among the Hop proteins is striking, with sequence identities ranging from 50 to 100% (Figure S1). This analysis too suggested that the current Hop and Hor classifications are not meaningful, with a subgroup of Hop proteins including SabA, BabA, and HopA displaying significantly strong identity shown in the top‐left corner of the heatmap, whereas all other Hop proteins are interdispersed with the Hor proteins at the opposite side of the map, including AlpA and OipA (Figure 3). The Hp_OMP domain of this overall collection of proteins shows the most similarity to the Hp_OMP domain of Family 3 Hom proteins, and thereafter to Family 2 Hof proteins.

Taken together with previously published observations (Alm et al., 2000), we conclude that the genus *Helicobacter* has a protein family defined by the Hp_OMP domain, where this domain represents at least a large segment of a transmembrane β‐barrel. Diversity within the family, possibly including the functions provided by each of the Hop, Hor, Hof, and Hom proteins, is achieved through the distinct passenger domains that each of these proteins projects onto the cell surface. We hypothesize that the minimalized sequence diversity in the β‐barrel domain provides a basis for a simplified BAM system to succeed in assembly of the diverse functional activities in the outer membrane proteome.

### 3.3 Characterization of the BAM complex in *H. pylori*

**Figure 4:**
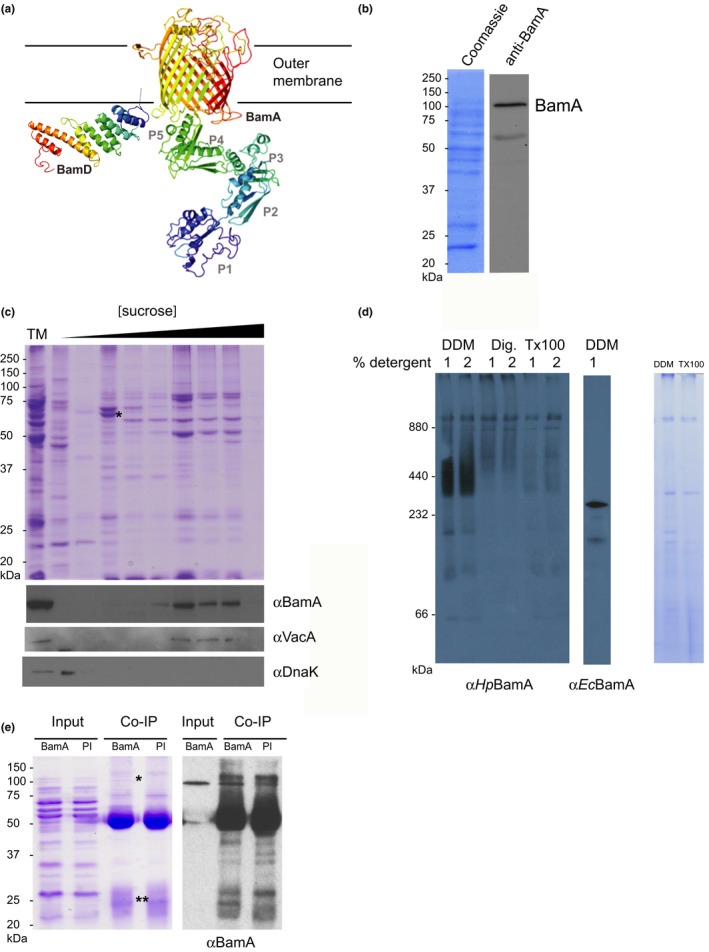
The BAM complex in *H. pylori*. (a) Phyre2 models for the structures of *Hp*BamA and *Hp*BamD. The POTRA domains are designated P1‐P5 and the predicted position of the outer membrane (OM) is indicated. The α‐helices of the tetratricopeptide repeated in BamD are evident and are colored from N‐terminal (violet) to C‐terminal (red). The N‐terminal cysteine residue of the processed BamD lipoprotein would position it at the inner surface of the outer membrane. (b) Total membranes were isolated from *H. pylori* and samples were subject to SDS‐PAGE and analyzed by either Coomassie blue staining or immunoblotting with anti‐*Hp*BamA. (c) Total membranes (TM) isolated from *H. pylori* were subjected to sucrose density gradient centrifugation. Fractions from the gradients were analyzed by SDS‐PAGE and Coomassie blue staining (upper panel) or immunoblotting with anti‐*Hp*BamA, anti‐VacA (outer membrane marker), anti‐DnaK (soluble protein marker). The “*” denotes an inner membrane protein TlpB, identified by mass spectrometry as a marker for the inner membrane fraction. (d) BN‐PAGE of *Hp*BAM complex in the presence of detergents DDM, Digitonin (Dig), and TritonX‐100 (Tx100) and a comparison with the *Ec*BAM complex solubilized in 1% DDM. SDS‐PAGE of solubilized membranes displaying difference in the proteome after treatment with 1%DDM or 1% Triton X‐100. (e) Coomassie stain (left panel) and immunoblot with anti‐HpBamA (right panel) of coimmunoprecipitation experiment. Input: Isolated total membranes; PI: control experiment performed with preimmune serum; BamA: experiment performed with αBamA. “*” denotes identification of BamA by immunoblot and mass spectrometry; “**” denotes identification of BamD by mass spectrometry

Previously, we developed Hidden Markov Models for comprehensive detection of subunits of the BAM complex (BamA, BamB, BamC, BamD, and BamE) and the TAM (TamA, TamB) (Anwari et al., 2012; Heinz & Lithgow, 2014; Heinz et al., 2015). Reanalysis of the data from these comprehensive searches demonstrated that there are no sequences corresponding to BamB, BamC or BamE, nor TamA or TamB in *H. pylori*. The *H. pylori* 26695 genome encodes a BamA (NP_207449.1) and BamD (NP_208169.1). Phyre predictions confirmed conserved structural features of these proteins to their *E. coli* counterparts. In *H. pylori,* BamA has five predicted POTRA domains and a C‐terminal 16‐stranded β‐barrel domain, whereas BamD is composed entirely of a series of tetratricopeptide repeats (Figure 4a).

In order to determine whether or not there are additional proteins present in the BAM complex of *H. pylori*, antibodies were raised to the BamA POTRA domain. The antibodies are specific and recognize a ~100 kDa protein in total membrane extracts from *H. pylori* after SDS‐PAGE analysis and in some instances a minor band indicative of BamA degradation (Figure 4b). The minor ~65 kDa protein was detected inconsistently in membrane preparations and is the correct size to represent the POTRA domain fragment.

Bacterial membrane extracts were prepared from *H. pylori* and subjected to sucrose density gradient fractionation. Immunoblotting of gradient fractions revealed BamA in the higher sucrose percentage of the gradient, a fractionating pattern similar to that observed for the outer membrane protein VacA (Olofsson et al., 2010). The position of the heat shock protein DnaK signifies that soluble nonmembrane proteins are concentrated in the first fraction of the gradient (Figure 4c). Further fractions were isolated from the gradient and analyzed by mass spectrometry. The most abundant protein (marked by a “*”) was identified as the inner membrane protein TlpB (Goers Sweeney et al., 2012), signifying the position of the inner membrane fraction in the gradient (Figure 4c). Western blots to localize BamA and VacA revealed that the outer membranes were present in the highest density fractions (Figure 4c).

To further characterize the BAM complex, membranes were solubilized in detergents for analysis by blue‐native gel electrophoresis (BN‐PAGE). This technique has been useful in identifying the size and modular nature of BAM complexes from other species, with studies predominantly using the detergent dodecyl maltoside (DDM) at concentrations above its critical micelle concentration (the CMC for DDM is 0.006% in 0.2 mol/L NaCl; Anwari et al., 2010; Webb et al., 2012). In *H. pylori,* a stable complex containing BamA migrates at an apparent molecular size of 440 kDa, irrespective of the concentration of DDM used to solubilize the membranes (Figure 4d). No BAM complex could be recovered when the membranes were extracted with other detergents, such as Triton X‐100 (Tx100) or digitonin (Dig), signifying that these are either insufficient to solubilize *H. pylori* membranes (Figure 4d). Figure 4d also shows that the size of the BAM complex from *H. pylori* is considerably larger than the BAM complex in *E. coli*, with the *E. coli* complex containing BamA, BamB, BamC, BamD, and BamE and having an overall size of ~240 kDa (Webb et al., 2012).

DDM‐solubilized membranes were then subject to coimmunoprecipitation to investigate protein‐protein interactions (Figure 4e). Attempts to validate the immunoprecipitation by Western blot analysis were ambiguous given reactivity of the secondary antiserum to the rabbit IgG used for the coIP. Analysis of the regions of the gel corresponding to ~100 kDa region of the gel and the ~26 kDa region of the gel was undertaken by mass spectrometry, comparatively with the equivalent region of the gel in the preimmune control experiment using preimune serum. The data indicate that both BamA and BamD were enriched: BamA with a score of 323 and comparative Student's *t*‐test difference in intensity between the BamA co‐IP and preimmune experiments of 3.8; BamD with a score of 72 and comparative intensity student *T*‐test of 5 (Figure S2 for full data analysis). This is consistent with the expectation that *Hp*BamD is a binding partner of *Hp*BamA. We also sifted the mass spectrometry data for potential novel partner proteins in the BAM complex in *H. pylori*. Most of the proteins identified are known to be highly abundant and present in the cytoplasm, and therefore must be considered unavoidable contaminants of the immunoprecipitation. Based on the values of the Score, sequence coverage and *T*‐test statistics, the mass spectrometry revealed nothing significant in the ~100 kDa region of the gel and only one protein in the ~26 kDa region of the gel that could be considered a candidate for further investigation. The protein, O25872_HELPY, is predicted to be an outer membrane lipoprotein, like BamD, but has a Pfam domain (PF03767) that would suggest it to be an acid phosphatase rather than a component of the BAM complex. O25872_HELPY is found in the genomes of other *Helicobacter* spp., but is also present (sequence identity >90%) in the genomes of many *Pseudomonas* spp. with the Pseudomonads being Gamma‐proteobacteria that have a characteristic BamABCDE complex (Anwari et al., 2012). In the absence of further data on O25872_HELPY we conclude conservatively that it is a 26 kDa protein that could be considered a candidate component of the BAM complex in *H. pylori*.

### 3.4 Characterization of HpBamA

To investigate the large apparent size *H.pylori* BAM complex in more detail, we set out to purify recombinant BamA and BamD. Despite all efforts, expression of native, full‐length BamA or BamD in *E. coli* was not possible, consistent with previous failures in the production of *H. pylori* proteins in the outer membrane and periplasmic environments of *E. coli* (Browning et al., 2015; Fischer et al., 1999; Paramasivam, Habeck, & Linke, 2012). As an alternative, we expressed the N‐terminal region of *Hp*BamA containing the predicted five POTRA repeats that should then behave as a soluble protein. Sequence alignment and tertiary structure prediction using Phyre2 predict the POTRA domains to be contained within residues 1‐469. SignalP predicts that the signal sequence is cleaved at Glu22/Asn23 (Figure S3) so a construct composed of residues Asn23 to Arg469 containing an N‐terminal, TEV‐cleavable His6‐tag was generated (herein call *Hp*BamA_POTRA_) for recombinant protein expression.

**Figure 5:**
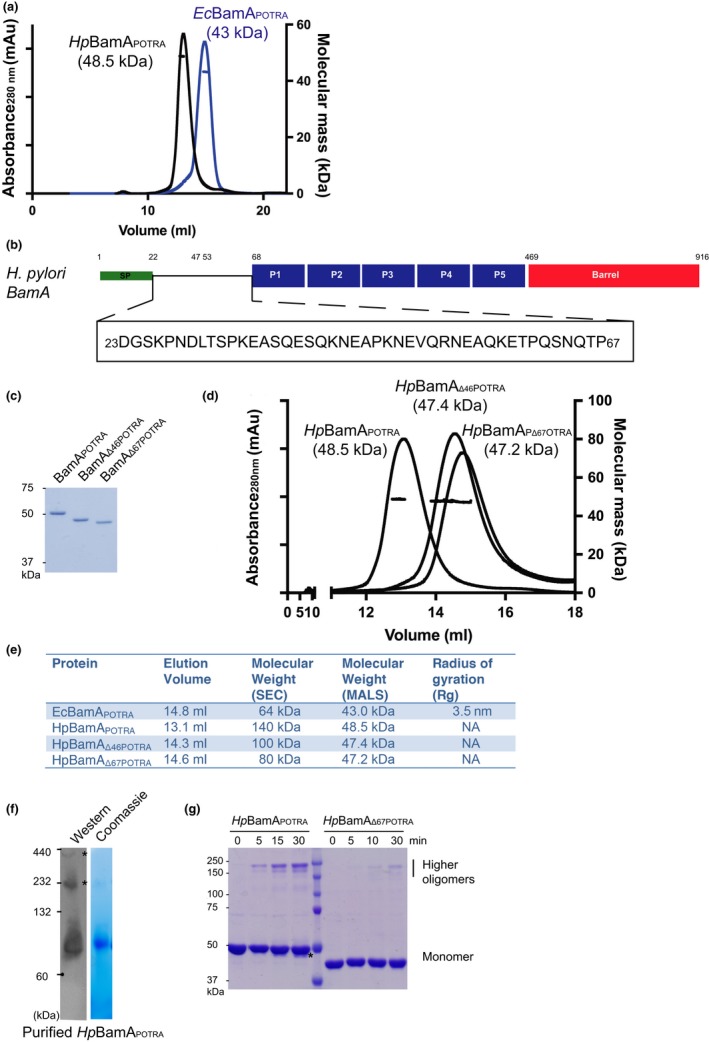
Characterization of recombinant *Hp*BamA POTRA domains. (a) The absolute molecular mass of purified recombinant proteins *Hp*BamA_POTRA_ and *Ec*BamA_POTRA_ were assessed using SEC‐MALs. (b) Schematic representation of *Hp*BamA highlighting the sequence of the N‐terminus extension (see FigureS3). (c) Purified *Hp*BamA_POTRA_ truncated proteins were characterized by SDS‐PAGE and Commassie blue staining. The migration positions of the molecular weight markers are shown. D. Comparison of *Hp*BamA_POTRA,_ *Hp*BamA_Δ46POTRA_, and *Hp*BamA_Δ67POTRA_ using SEC‐MALs. The details of the analysis are described in the Methods section. (e). Summary of SEC‐MALs data for the various POTRA domain constructs. (f) Purified *Hp*BamA_POTRA_ was analyzed by BN‐PAGE and immunoblotting. The western was probed with anti‐*Hp*BamA antibodies, indicating that *Hp*BamA_POTRA_ runs larger than its monomeric size of 48.5 kDa indicative of its elongated shape, and highlights the presence of oligomers (asterisks). The migration positions of molecular weight standards are indicated. (g) The oligomerization of *Hp*BamA_POTRA_ was captured by BS3 cross‐linking. The purified protein was incubated with cross‐linker for the indicated time, and then analyzed by SDS‐PAGE and Coomassie blue staining. Asterisk denotes *Hp*BamA_POTRA_ that has been truncated at Asn53, as judged by N‐terminal sequencing

To assess the size of *Hp*BamA_POTRA_ in solution, SEC‐MALS was used. The MALS data indicated an absolute mass in solution of 48.5 kDa for *Hp*BamA_POTRA_ (consistent with its predicted molecular mass of 51 kDa), whereas SEC data showed that the protein eluted much earlier than expected with an approximated size of ~140 kDa (Figure 5a). The difference in mass between MALS and SEC is reconciled only if *Hp*BamA_POTRA_ adopts an elongated shape, far from being globular. The shape of the POTRA domains from *E. coli* is quite distinct, in that this domain construct behaves as predicted from crystal structures (Kim et al., 2007): the *E. coli* BamA_POTRA_ (predicted molecular mass 45 kDa) elutes as an extended monomer on SEC (~65 kDa) with an absolute mass of 43 kDa (Figure 5a).

Sequence analysis of BamA from *H. pylori* and comparison to *Ec*BamA revealed an N‐terminal extension prior to the conserved five POTRA domains in *Hp*BamA (Figure 5b, S3). Secondary structure predictions of this extension suggested it to be natively disordered. In addition, N‐terminal sequencing of a degradation product observed in the purified *Hp*BamA_POTRA_ demonstrated it was truncated at Asn53 suggesting that this *Helicobacter*‐specific region is amenable to degradation (Figure 5b,g). We hypothesized that this N‐terminal extension might be responsible for the retardation of *Hp*BamA_POTRA_ observed by SEC and that with its removal *Hp*BamA_POTRA_ would behave similar to its analogous *Ec*BamA_POTRA_. To test this, truncation mutants of the N‐terminus up to residues 46 and 67, namely *Hp*BamA_Δ46POTRA_ and *Hp*BamA_Δ67POTRA_, were purified and their molecular mass assessed by SEC‐MALS (Figure 5c,d and e). *Hp*BamA_Δ67POTRA,_ which lacks the complete N‐terminal extension, eluted as an extended monomer (with an apparent molecular weight of 80 kDa) and an absolute molecular mass of 47.2 kDa (Figure 5d); very similar to the elution profile of *Ec*BamA_POTRA_ (Figure 5a). *Hp*BamA_Δ46POTRA_ in which only half of the extension is removed, eluted mid‐way between *Hp*BamA_POTRA_ and *Hp*BamA_Δ67POTRA_ at ~100 kDa by SEC (Figure 5d), significantly larger than its suggested molecular mass in solution of 47.4 kDa (Figure 5c,d and e). These results indicate that the N‐terminal extension in *Hp*BamA plays a significant role in the elongated shape of *Hp*BamA. Consistent with this conclusion, the radius of gyration could not be calculated with any confidence for any of the *H.pylori* BamA proteins, whereas *Ec*BamA_POTRA_ displayed a radius measurement of 3.5 nm consistent with a more globular shape.

Regions of native disorder mediate protein–protein interactions and, given the large size of the BAM complex observed by BN‐PAGE, recombinant *Hp*BamA_POTRA_ was examined by BN‐PAGE. Coomassie staining revealed that *Hp*BamA_POTRA_ migrated much slower than its expected monomeric size (Figure 5f). To independently address the possibility of oligomerization, purified *Hp*BamA_POTRA_ was incubated with the chemical cross‐linker BS^3^ to covalently capture any protein–protein interactions over time, revealing *Hp*BamA_POTRA_ oligomers (Figure 5g). To verify whether this multimerization was a result of the N‐terminal extension, the same experiment was performed with recombinant *Hp*Bam_Δ67POTRA_, limiting its multimerization. In summary, these results suggest *Hp*BamA is elongated in shape, and is capable of intermolecular interactions via its N‐terminal extension.

## 4 DISCUSSION

Over the 65,000 year long history of cohabitation between *H. pylori* and humans evolutionary pressure has driven changes in the BAM complex—and its substrates—to maximize protein assembly in the face of significant genome reduction. This contrasts to the scenario seen in the model bacterium *E. coli*, where a BAM complex composed of five subunits coordinates with the further two subunits of the TAM to drive the assembly of OMPs. Deletion of *bamA* or *bamD* is lethal in *E. coli*, making BamA and BamD the essential core of the BAM complex. This study shows that the BAM complex in *H. pylori* maintains this essential core, in common with other proteobacteria. There are, however, distinguishing features in the BAM complex of *H. pylori*.

Although other components may be present in the BAM complex of *H. pylori*, we were unable to unequivocally identify any. Perhaps the most promising candidate was the protein designated O25872_HELPY, predicted to be a lipoprotein and therefore possibly located in the outer membrane. Pfam domain characterization suggests that the protein functions instead as an acid phosphatase, but it remains formally a possibility that *H. pylori* might have coopted this protein as a *Helicobacter*‐specific component of the BAM complex. The alternative would seem to be that in *H. pylori*, the BAM complex is composed of only BamA and BamD.

Irrespective of any potential *Helicobacter*‐specific components, analysis of *H. pylori* outer membrane extracts revealed that BamA exists in a distinctly large structure. In order to form such a large complex, multiple copies of BamA and BamD subunits would need to oligomerize. Direct evidence of a mechanism for oligomerization mediated through an N‐terminal extension in the POTRA domains of BamA was established by both BN‐PAGE and by chemical cross‐linking. The propensity for this multimerization was diminished after the removal of the N‐terminal extension from BamA. Interestingly, a similar extension was observed in the BamA homologue of *Helicobacter acinonychi* (Figure S3), thought to have evolved by a host jump between large felines (cheetahs) and humans (Eppinger et al., 2006). Thus, the N‐terminal extension of BamA appears to be a feature of human adaptation.

The evolutionary pressure for genome reduction may have driven a minimalist scenario for OMP biogenesis but, conversely, the pathogen's environment requires diversity in the function of its outer membrane. We propose that a key contributing factor to reconcile these opposing evolutionary forces comes in the form of a cut‐and‐paste mechanism for providing OMP diversity through the use of the Hp_OMP β‐barrel domain. This region displays relatively little sequence diversity in these OMPs that is, in the part that is bound and folded by the BAM complex. Diversity of function is instead provided via a wide range of “passenger” domains being appended to many of these β‐barrel domain such as the VacA autotransporters and Hp_OMP families. In particular, the Hp‐OMP families appear to represent a new class of Type 5 secretion systems. This suggestion has been made previously (Alm et al., 2000; Voss et al., 2014)), and our findings here add further support to the speculation. The topography whereby an extracellular passenger domain is displayed via a transmembrane β‐barrel assembled into the cell surface is the defining feature of a Type 5 secretion system. Currently there are at least six subtypes (Bleves et al., 2010; Fan, Chauhan, Udatha, Leo, & Linke, 2016; Heinz et al., 2016; Leo, Oberhettinger, Schutz, & Linke, 2015) distinguished by the β‐barrel domain used as the protein translocation device: autotransporters (Type Va), two‐partner secretion systems (Type Vb), trimeric autotransporters (Type Vc), patatins (Type Vd), inverse‐autotransporters (Type Ve) and two‐partner inverse‐autotransporters (Type Vf). The Hp_OMP families of Hop and Hor proteins would constitute the first subtype of the Type 5 secretion system to be exclusive to a single genus of bacteria.

## CONFLICT OF INTEREST

None declared.
