# Supplementary material for: Reductive evolution in outer membrane protein biogenesis has not compromised cell surface complexity in Helicobacter pylori
Source: Microbiologyopen. 2017 Oct 21;6(6):e00513. doi: 10.1002/mbo3.513 (PMC5727368; doi:10.1002/mbo3.513)
Supplement: Supplementary file 1 [file MBO3-6-na-s001.pdf]

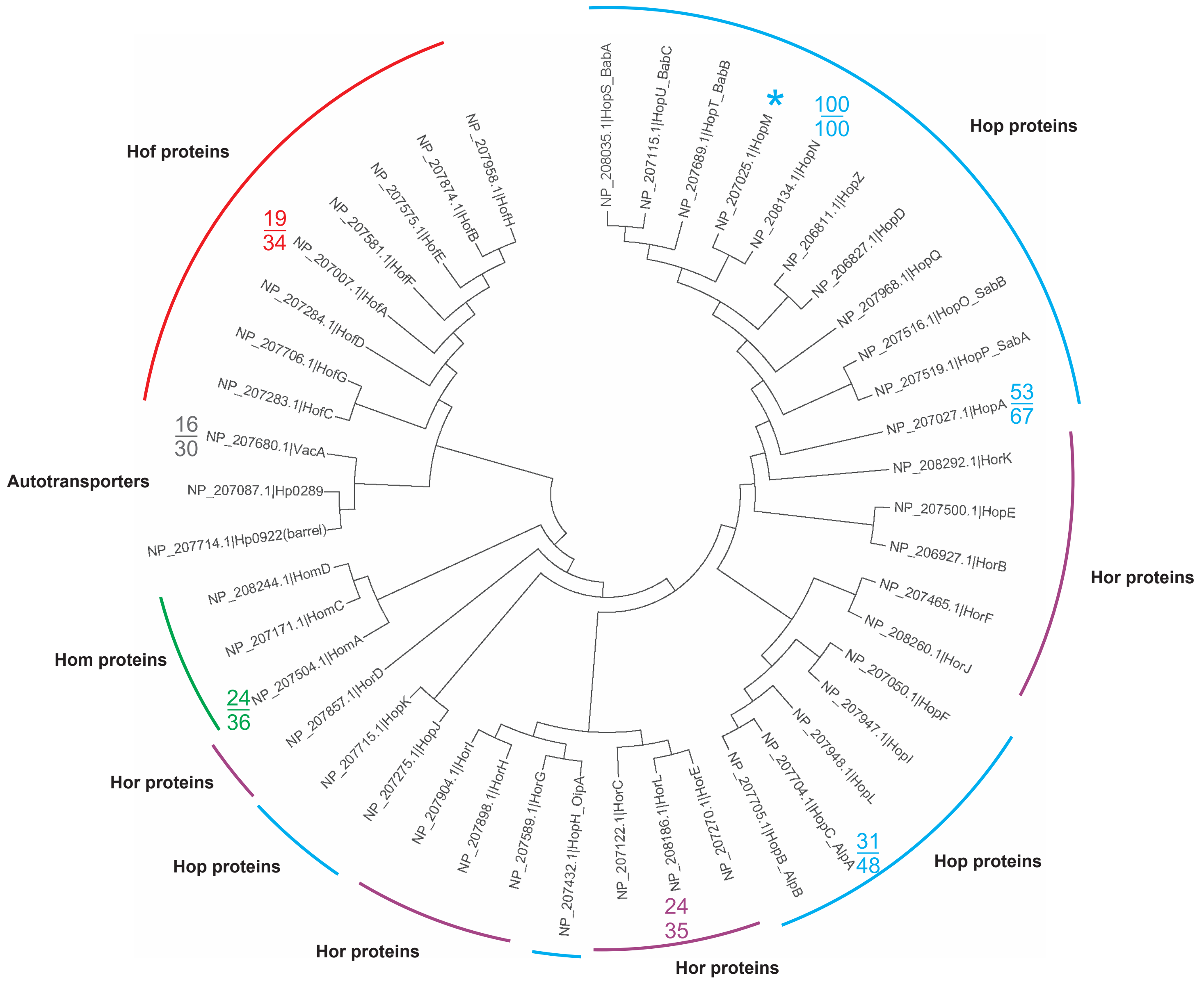

Figure S1. Phylogenetic tree of the barrel domain. Phylograph of the C-terminal 250 residues from the Hop/Hor, Hof, Hom and also the autotransporter families. The tree was constructed with MEGA6 using ClustalW alignment and Neighbour Joining method (partial deletion). To give a sense of the scale of the tree, relative to HopM (blue asterix) the sequence identity for several other proteins is indicated; the upper number is sequence identity, the lower number is sequence similarity. The pairwise identities and similarities for these C-terminal sequences were calculated with EMBOSS Needle protein refinement tool.

Supplementary Figure S1

Comparison between band excised at ~100kDa from BamA Co-IP and Preimmune denoted * in Figure 4E.

Protein IDs	Name	Mol. weight [kDa]	Peptides	Sequence coverage [%]	Score	Intensity	MS/MS count	Student's T-test Difference LFQ intensity 1_LFQ intensity 2	LFQ intensity 1	LFQ intensity 2
sp|O06913|FRDA_HELPY	Fumarate reductase flavoprotein subunit	80.12	6	9	40.566	4427200	6	6.48215294	22.07796	15.59581
sp|O25249|PGBA_HELPY	Plasminogen-binding protein PgbA	52.655	1	2.2	8.2185	493300	1	2.17125893	18.91211	16.74085
sp|O25369|BAMA_HELPY	BamA	102.67	33	41.7	323.31	416300000	117	3.80603981	28.29312	24.48708
sp|O25534|PGBB_HELPY	Plasminogen-binding protein PgbB	61.899	4	9.8	22.544	1344500	3	4.12577057	20.35864	16.23287
sp|P0A0R3|CH10_HELPY	GroES	12.991	3	23.7	23.562	17348000	6	2.94522858	18.67793	15.7327
sp|P42383|CH60_HELPY	GroEL	58.263	7	17.2	44.749	5205900	11	2.10700798	20.91108	18.80407
sp|P56098|RODA_HELPY	Peptdoglycan glycosyltransferase MrdB	43.136	1	2.1	6.2606	27375000	2	2.61545372	18.87217	16.25671
sp|P56155|PYRF_HELPY	OMP decarboxylase	25.383	2	9.3	11.809	1579900	3	2.57570553	17.67858	15.10287
sp|P56452|SYA_HELPY	Alanine-tRNA ligase	94.699	6	7.7	40.318	2167900	6	4.02078819	20.8659	16.84511
sp|Q09066|UREG_HELPY	Urease accessory protein UreG	21.955	4	20.1	29.076	6364700	8	3.84436989	17.52507	13.6807
tr|O24870|O24870_HELPY	Outer membrane protein (Omp2)	77.664	15	30.2	320.66	171550000	36	4.29235649	27.04625	22.75389
tr|O25006|O25006_HELPY	hyptothetical protein	20.69	1	9.8	17.819	0	1	2.48169327	19.23108	16.74938
tr|O25076|O25076_HELPY	hyptothetical protein	20.385	4	25	25.497	4348700	4	2.14505196	18.36246	16.21741
tr|O25091|O25091_HELPY	Outer membrane protein (Omp10)	28.766	2	8.7	12.937	2842800	2	3.01241302	16.2917	13.27928
tr|O25286|O25286_HELPY	3-ketoacyl-acyl carrier protein reductase (FabG)	26.668	5	26.3	51.847	9031100	5	2.52906513	17.57134	15.04228
tr|O25543|O25543_HELPY	Iron-regulated outer membrane protein (FrpB)	88.524	8	15.9	45.811	11477000	10	8.07187557	23.06707	14.99519
tr|O25950|O25950_HELPY	Iron(III) dicitarte transport protein FecA	94.826	9	13.4	66.203	6822000	13	6.49793339	22.48962	15.99168
tr|O26064|O26064_HELPY	Ubiquinol cytochrome c oxidoreductase, cytochrome b subunit (FbcH)	47.51	2	5.1	11.569	389340	1	2.61854172	17.62421	15.00567

Comparison between band excised at ~26kDa from BamA Co-IP and Preimmune denoted ** in Figure 4E.

Protein IDs	Name	Mol. weight [kDa]	Peptides	Sequence coverage [%]	Score	Intensity	MS/MS count	Student's T-test Difference LFQ intensity 3_LFQ intensity 4	LFQ intensity 3	LFQ intensity 4
sp|O25766|TRMD_HELPY	M1G-methyltransferase	25.879	1	4.8	9.6778	607530	1	2.45566845	16.67204	14.21637
sp|O25858|NUOI_HELPY	NADH dehydrogenase I subunit I	24.715	4	17.3	26.285	3515800	5	3.41375828	19.20483	15.79108
sp|O25930|BAMD_HELPY	BamD	26.256	6	32.3	72.352	16444000	8	5.04290009	21.38147	16.33857
sp|O26107|Y1588_HELPY	UPF0174 protein HP_1588	28.417	3	13	19.621	2022300	2	3.79194546	18.40699	14.61504
sp|P42383|CH60_HELPY	GroEL	58.263	7	17.2	44.749	5205900	11	4.08973217	19.60485	15.51512
sp|P55992|GYRB_HELPY	DNA gyrase subunit B	87.366	3	4.1	17.451	771620	3	2.2074461	16.24494	14.0375
sp|P56029|RL1_HELPY	50S ribosomal protein L1	25.266	6	27.4	95.059	12324000	6	4.92894173	21.01441	16.08547
sp|P56032|RL4_HELPY	50S ribosomal protein L14	24.023	2	14.9	16.24	5004400	2	4.36925507	19.7142	15.34494
sp|P56034|RL6_HELPY	50S ribosomal protein L16	19.486	3	21.3	18.563	3311100	4	2.35906792	18.93275	16.57368
sp|P56098|RODA_HELPY	Peptdoglycan glycosyltransferase MrdB	43.136	1	2.1	6.2606	27375000	2	5.61960888	21.15116	15.53155
sp|P56106|PYRH_HELPY	Uridylate kinase	26.172	4	22.1	32.026	6283700	3	3.99811935	20.0426	16.04448
sp|Q09065|UREF_HELPY	Urease accessory protein UreF	28.619	5	23.6	46.511	8294400	5	5.22021389	20.44314	15.22292
tr|O25076|O25076_HELPY	hypothetical protein	20.385	4	25	25.497	4348700	4	3.10971069	19.51156	16.40185
tr|O25633|O25633_HELPY	conserved hypothetical integral membrane protein	30.442	7	29.6	83.027	27290000	12	7.02507305	22.15153	15.12645
tr|O25834|O25834_HELPY	hypothetical protein	22.149	1	3.8	7.9672	169370000	3	2.2820282	16.83363	14.5516
tr|O25872|O25872_HELPY	conserved hypothetical secreted protein	26.297	4	15.7	31.941	14334000	6	5.06376648	21.2324	16.16863
tr|O25974|O25974_HELPY	hypothetical protein	99.278	1	1.8	8.5724	635270000	7	9.32419968	25.92059	16.59639
tr|O34945|O34945_HELPY and ;tr|O34491|O34491_HELPY	IS605 transposase (TnpB)	49.262	1	2.8	6.4876	7291300	1	4.30545712	20.25718	15.95173

Figure S2. Mass spectrometry comparitive analysis of BamA co-immunoprecipitation bands. Coomassie-stained bands from a BamA co-IP pull-down and respective preimmune pull-down were analysed by mass spectrometry and the peptide outputs compared. Bands at ~100kDa pertaining to BamA (BamA co-IP and preimmune sample intensities labeled 1 and 2 respectively) along with a band at ~26 kDa (BamA co-IP and preimmune sample intensities labeled 3 and 4 respectively) were analyzed (Fig. 4E) and then the difference in intensities compared using a Student's T-test (a difference >2 is considered significant).

Supplementary Figure S2

```
H.pylori          MKKLILSSLVFACINTSVEALENDGSKPNDLTSPKEASQESQKNEAPKNEVQRNEAQKET     58
H.acinonychis     +KRIILTSLVFVYFNTSVEALENNGSKPNGVASQKETEAP-------K----------N     41
E.coli            MAMKKLLIASLLFSSATVYG----------------------------------------     20
                   :  :* ::::   :::*::

H.pylori          PQSNQTPKEMKVKSISYVGLSYMSDMLANEIVKIRVGDIVDSKKIDTAVLALFNQGYF     118
H.acinonychis     ETTNETPKEIKVKSVSYVGLSYMSDMLANEIVKIRVGDIVDSKKIDTAVLALFNQGYF     100
E.coli            ------AEGFVVKDIHFEGLQRVAVGAALLSMPVRTGDTVNDEDISNTIRALFATGNF      72
                         : : **.: : **. ::   *   : :*.*. *:.: *..:: ***  * *
```

Figure S3. The N-terminal sequence extension in *Helicobacter pylori* BamA.
The N-terminal sequences of BamA from Epsilon-proteobacteria (*Helicobacter spp*) and the model Gamma-proteobacterium (*E. coli*) are aligned to detail the presence of an N-terminal extension immediately after the signal sequence present in *H. pylori* and *H. acinonychis*. The "+" indicates ambiguity in the start codon for the various *H. acinonychis* genome sequences, underscored sequence represents the signal sequence predicted by SignalP. The sequence alignment was performed on the full protein sequences using the Clustal Omega multiple sequence alignment tool, only the N-terminal segment of the multiple sequence alignment is shown. "*" denotes identity in all three sequences, ":" denotes identity in 2/3 sequences, "." denotes conservative substitutions.

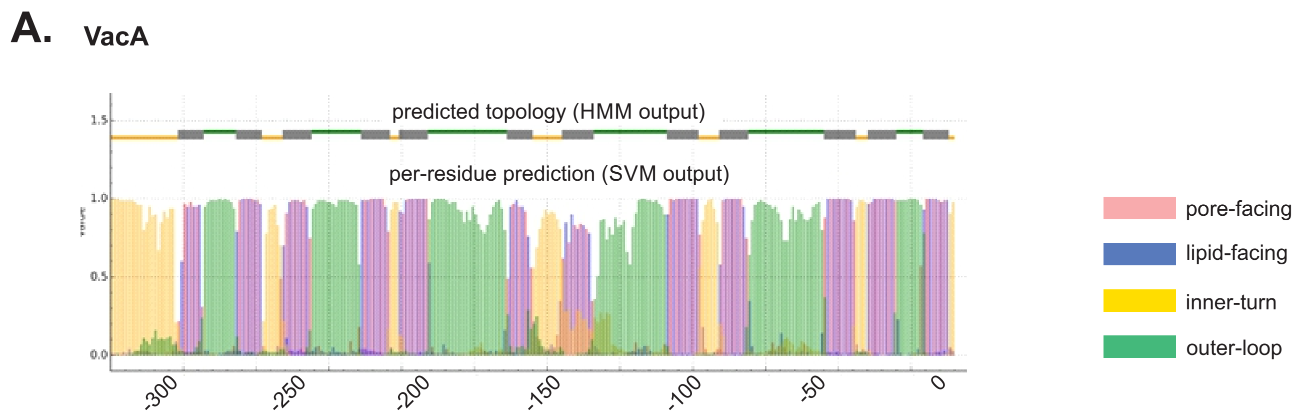

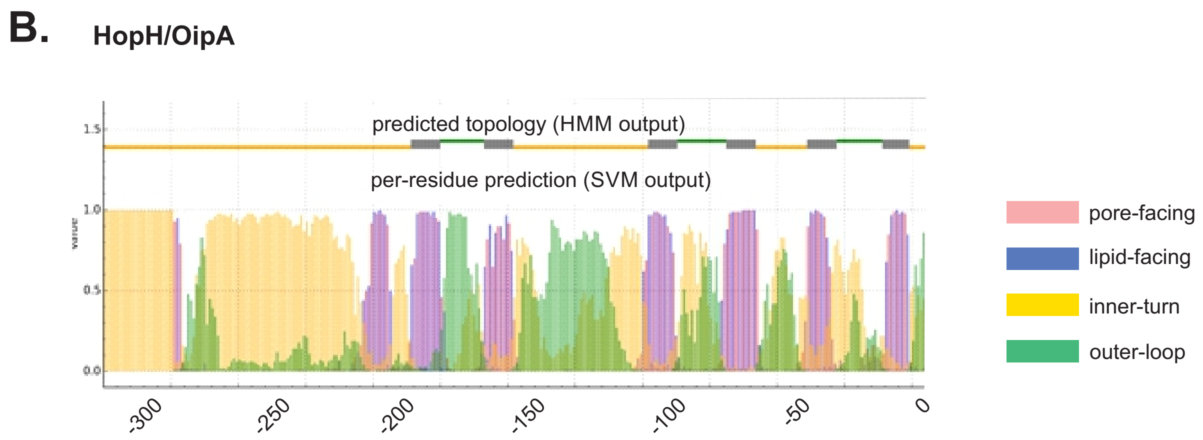

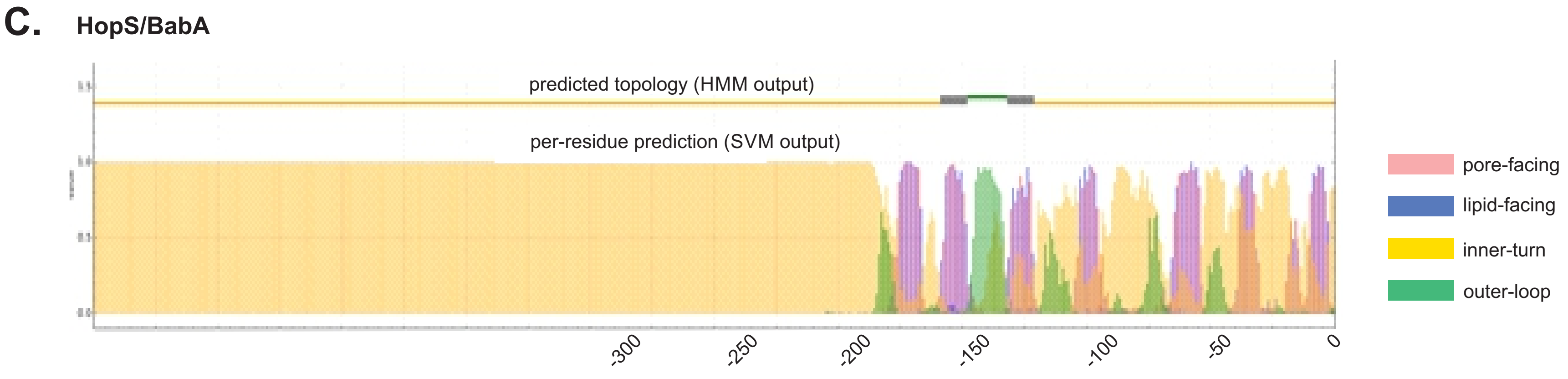

Supplementary Fig. S4 Reproduction of β-barrel prediction outputs.
A. The prediction for the C-terminal region of VacA sequenceas depicted in Fig. 1D, shows the residue-by-residue ("per residue" values) mapping for pore-facing/lipid-facing values and for inner-turn and outer-loop predictions in the lower panel. Above this, the representation for the predicted topology according to the standard cut-off scores, which may or may not be appropriate for *Helicobacter* outer membrane proteins (see main text). **B.** Data from Fig. 2B for HopH/OipA. **C.** Data from Fig. 2B for HopS/BabA.

Supplementary Figure S4
